# Interstitial lung abnormalities in a large clinical lung cancer screening cohort: association with mortality and ILD diagnosis

**DOI:** 10.1186/s12931-023-02359-9

**Published:** 2023-02-14

**Authors:** Avignat S. Patel, Ezra Miller, Shawn M. Regis, Gary M. Hunninghake, Lori Lyn Price, Melissa Gawlik, Andrea B. McKee, Kimberly M. Rieger-Christ, Victor Pinto-Plata, Timothy N. Liesching, Christoph Wald, Jeffrey Hashim, Brady J. McKee, Lee Gazourian

**Affiliations:** 1 Division of Pulmonary and Critical Care Medicine, Department of Medicine, Lahey Hospital and Medical Center, Burlington, MA 01805 USA; 2 Tufts University School of Medicine, Boston, MA 02111 USA; 3 Division of Radiation Oncology, Department of Medicine, Lahey Hospital and Medical Center, Burlington, MA 01805 USA; 4 Division of Pulmonary and Critical Care Medicine, Department of Medicine, Brigham and Women’s Hospital, Boston, MA 02115 USA; 5 Harvard Medical School, Boston, MA 02115 USA; 6 Institute for Clinical Research and Health Policy Studies, Tufts Medical Center, Boston, MA 02111 USA; 7 Tufts Clinical and Translational Science Institute, Tufts University, Boston, MA 02111 USA; 8 Quality and Safety, Lahey Hospital and Medical Center, Burlington, MA 01805 USA; 9 Translational Research, Lahey Hospital and Medical Center, Burlington, MA 01805 USA; 10 Department of Radiology, Lahey Hospital and Medical Center, Burlington, MA 01805 USA

**Keywords:** Interstitial lung abnormalities, Interstitial lung disease, Idiopathic pulmonary fibrosis, Lung cancer screening

## Abstract

**Background:**

Interstitial lung abnormalities (ILA) are CT findings suggestive of interstitial lung disease in individuals without a prior diagnosis or suspicion of ILD. Previous studies have demonstrated that ILA are associated with clinically significant outcomes including mortality. The aim of this study was to determine the prevalence of ILA in a large CT lung cancer screening program and the association with clinically significant outcomes including mortality, hospitalizations, cancer and ILD diagnosis.

**Methods:**

This was a retrospective study of individuals enrolled in a CT lung cancer screening program from 2012 to 2014. Baseline and longitudinal CT scans were scored for ILA per Fleischner Society guidelines. The primary analyses examined the association between baseline ILA and mortality, all-cause hospitalization, and incidence of lung cancer. Kaplan–Meier plots were generated to visualize the associations between ILA and lung cancer and all-cause mortality. Cox regression proportional hazards models were used to test for this association in both univariate and multivariable models.

**Results:**

1699 subjects met inclusion criteria. 41 (2.4%) had ILA and 101 (5.9%) had indeterminate ILA on baseline CTs. ILD was diagnosed in 10 (24.4%) of 41 with ILA on baseline CT with a mean time from baseline CT to diagnosis of 4.47 ± 2.72 years. On multivariable modeling, the presence of ILA remained a significant predictor of death, HR 3.87 (2.07, 7.21; p < 0.001) when adjusted for age, sex, BMI, pack years and active smoking, but not of lung cancer and all-cause hospital admission. Approximately 50% with baseline ILA had progression on the longitudinal scan.

**Conclusions:**

ILA identified on baseline lung cancer screening exams are associated with all-cause mortality. In addition, a significant proportion of patients with ILA are subsequently diagnosed with ILD and have CT progression on longitudinal scans.

*Trial registration number*: ClinicalTrials.gov; No.: NCT04503044.

**Supplementary Information:**

The online version contains supplementary material available at 10.1186/s12931-023-02359-9.

## Background

Interstitial lung abnormalities (ILA) are CT scan findings suggestive of interstitial lung disease (ILD). These can be observed in individuals who have no prior diagnosis or suspicion of (ILD) [1–3]. An association between ILA and important clinical outcomes has been demonstrated, including mortality[4–6] and lung cancer incidence [7, 8]. A subgroup of those with ILA may have undiagnosed ILD, including progressive fibrotic ILD [9]. Identifying ILA may allow for an earlier stage diagnosis of ILD and may help to maximize the benefit of anti-fibrotic therapies that have been developed in the last decade [9–13].

Progressive fibrotic ILD is among the most clinically significant forms of ILD with respect to morbidity and mortality [14–16]. Idiopathic pulmonary fibrosis (IPF), the most common progressive fibrotic ILD, has a median survival of only 3–4 years [17, 18]. Despite the often rapid clinical decline, delays in the recognition and diagnosis of ILD, specifically IPF, are commonplace with a median time to diagnosis of 1–3 years after symptom onset [19].

Evaluating CT lung cancer screening (CTLS) cohorts may provide an opportunity to identify ILA and undiagnosed ILD. The individuals in these cohorts are high-risk for ILD by virtue of their smoking history, and most are followed over a period of years which can capture progression. In this study, we evaluated the prevalence of ILA, the associations with mortality, hospitalization and lung cancer, and the incidence and timing of subsequent ILD diagnosis, including IPF, among participants in our institution’s clinical CTLS program.

## Methods

### Study population

We conducted a retrospective, single-center study approved by the institutional review board. All clinical CTLS exams performed from January 1^st^, 2012, through September 30^th^, 2014, on participants who had an in-network primary care physician (n = 1703) were included. This enrollment period was chosen to allow for a 5-year follow-up time period from the last baseline scan. The dataset was closed on September 30^th^, 2019, except for data collection on pulmonary referral and ILD diagnosis, which was closed on October 31^st^, 2020. To qualify for our study, individuals had to satisfy the National Comprehensive Cancer Network (NCCN) Guidelines® Lung Cancer Screening Version 1.2012 high-risk criteria for lung cancer. Enrollees were asymptomatic and had a physician order for CTLS, were free of lung cancer for ≥ 5 years, and had no known metastatic disease [20]. Individuals with a documented history of ILD were excluded.

Follow-up scans used to evaluate ILA progression were designated by annual screening rounds with T0 representing the initial scan and T4 representing the 5th round of screening. Each round of screening ended with a negative scan (Lung-RADS® 1 or 2) and the next round of screening was determined by the first screening exam at least 11 months (335 days) after that negative scan. Individuals found to have a malignancy were censored at the time of first positive scan. Those with T4 screening scans were scored for progression (n = 652).

### Clinical variables

Baseline demographic and clinical variables, including age, sex, ethnicity, BMI, smoking history, were collected prospectively as part of the CTLS program and stored in a centralized data repository as previously described [21, 22]. Additional clinical variables, including cancer, mortality, cause of death, date of pulmonary referral, reason for pulmonary referral, and ILD diagnosis, not already available in this data repository, were collected retrospectively by manual review of the electronic medical record or pulled directly from the EMR and stored utilizing a custom-designed database (FileMaker ProVersion 11; Filemaker Inc, Santa Clara, California). Hospital admissions were collected using Lahey administrative coding data with hospitalization defined as first hospitalization after the baseline scan.

### CT analysis

Clinically acquired CTLS examinations were performed on ≥ 64-row multidetector CT scanners (LightSpeed VCT and Discovery VCT [GE Medical Systems, Milwaukee, Wisconsin]; Somatom Definition [Siemens AG, Erlangen, Germany]; iCT [Philips Medical Systems North America, Cambridge, Massachusetts]) at 100 kV and 30 to 100 mA, depending on the scanner and the availability of iterative reconstruction software. Axial images were obtained at 1.25- to 1.5-mm thickness with 50% overlap and reconstructed with both soft tissue and lung kernels [23].

CT images were qualitatively scored retrospectively for the presence and nature of ILA utilizing Philips PACS and clinical grade monitors for image review. Scoring was performed independently by two thoracic radiologists (B.J.M, J.H). Scores that were discordant between the two radiologists were subsequently scored by a third reader—a pulmonologist with expertise in ILD (L.G.). Descriptors used for ILA were defined based on the Fleischner Society position paper [3]. The presence of ILA was scored as Yes/No/Indeterminate. ILA features that were scored include: A) nondependent ground glass, B) reticular abnormalities, C) traction bronchiectasis, D) honeycombing, and E) cysts. Indeterminate ILA was defined as focal or unilateral ground glass opacity, focal or unilateral reticulation, and patchy ground glass opacity (< 5% of lung) [1].

#### Usual interstitial pneumonia

**Fig. 1:**
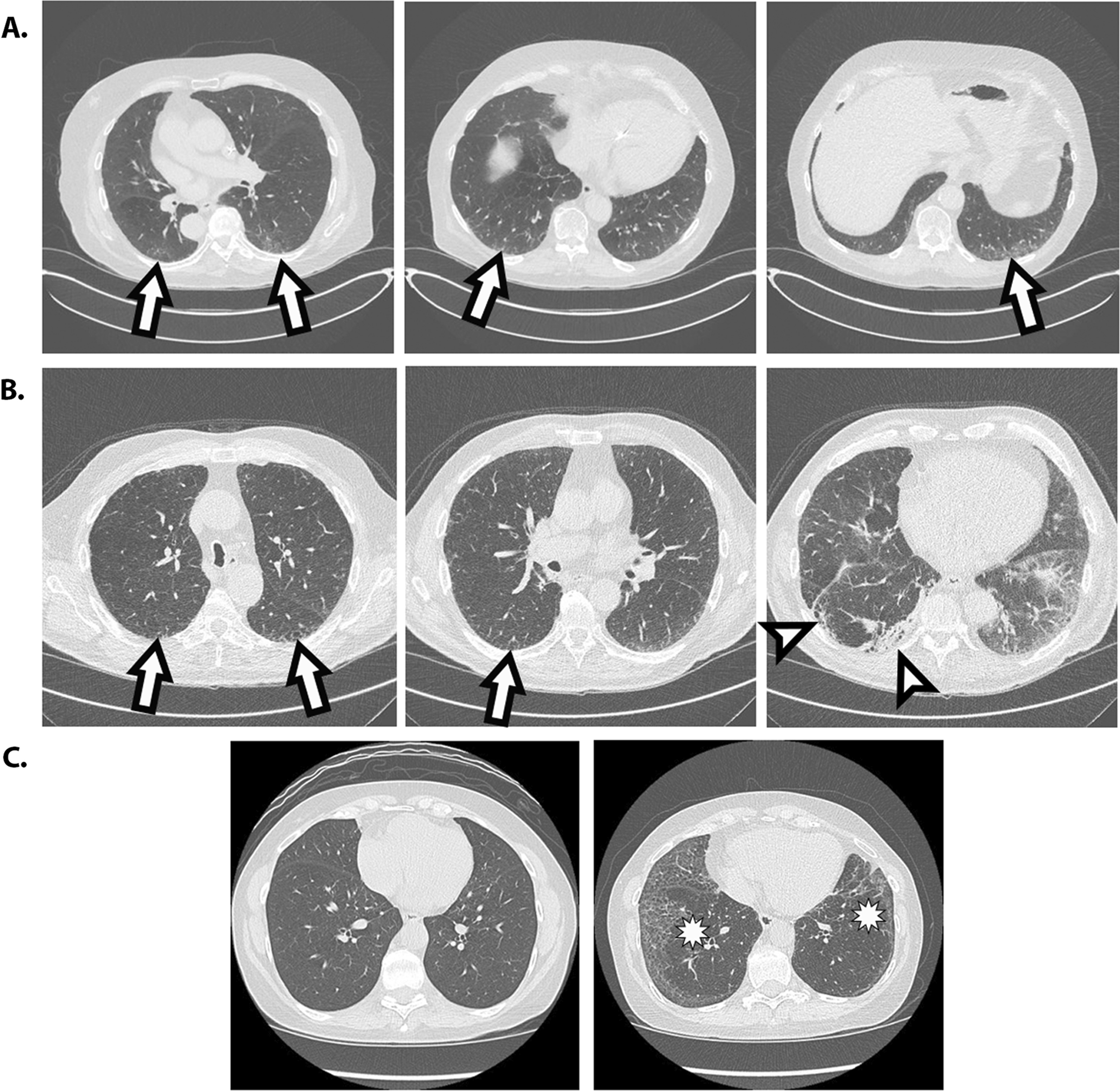
**A** Representative CT images from individual with ILA (arrows denote reticular opacities). **B** Representative CT images from individual with ILA, further characterized as probable UIP CT pattern (arrows denote subpleural reticular opacities, arrowheads denote traction bronchiectasis). **C** CT images demonstrating development of fibrotic ILA (asterisk) in a T4 scan (right) compared to no ILA at baseline (left)

ILA were further classified for usual interstitial pneumonia (UIP) (typical, probable, indeterminate, alternate diagnosis) based on ATS/Fleischner Criteria [24, 25]. Figure 1 depicts examples of individuals with ILA (A) and ILA meeting probable UIP criteria (B).

#### Progression

The subset of individuals who had T4 screening scans were independently scored as above, and in addition, were compared to their baseline (T0) scans and scored for progression: stable, improved, and progressed. Figure 1C illustrates an example of an individual with ILA progression.

### Statistical analysis

The ILA prevalence at baseline was described for the full cohort using frequencies and percentages by descriptor. Both presence and absence of ILA, as well as phenotypes, were described for the entire cohort. Progression of ILA, defined as worsening of existing ILA or incidence of ILA over 4 years, was described for the subset with T4 scans.

The primary analyses examined the association between baseline indeterminate ILA and ILA (presence/absence) and time to mortality, time to first hospitalization, and time to development of lung cancer in the full cohort. Kaplan–Meier plots were generated to visualize the associations between ILA variables and lung cancer, hospital admission and all-cause mortality. Cox regression proportional hazards models were used to test for this association in both univariate and multivariable models. The multivariable model was adjusted for age, sex, BMI, smoking status and pack years exposure; for the cox regression proportional hazards model for all-cause mortality, age was included as a time-dependent covariate.

Secondary analyses included Cox regression models to examine the association between phenotypes of ILA as the independent variables (separately) with outcomes of time to first hospitalization, lung cancer, and mortality adjusted for age, sex, BMI, smoking status and pack years exposure.

The Kappa (κ) value was calculated for agreement between first and second readers for the baseline scan. Interobserver agreement was categorized as poor (κ = 0–0.20), fair (κ = 0.21–0.40), moderate (κ = 0.41–0.60), good (κ = 0.61–0.80), or excellent (κ = 0.81–1).

To adjust for multiplicity, Bonferroni correction was utilized for our three primary outcomes and p-value significance was set at < 0.017. Otherwise, significance levels were set at a p-value ≤ 0.05. All statistical analyses were performed using STATA14.1 software (StataCorp LLC, College Station, TX).

## Results

A total of 2,560 individuals were enrolled in CT lung cancer screening between January 1st, 2012, and September 30th, 2014. Those enrolled with no outcomes were censored on September 30th, 2019, or time of last follow-up, which ever came first, and the average follow-up period was 5.67 ± 1.59 years. 857 were excluded because their primary care providers were outside of the network (Additional file 2: Table S1). Four (0.23%) were excluded because they carried a pre-existing ILD diagnosis. The remaining 1699 were included in our analysis (Additional file 1: Fig. S1). For those included, the mean age was 62.6 ± 6.2 years, 956 (56.3%) were male, and 1,670 (98.3%) white. 982 (57.8%) had visually identified emphysema. 776 (45.7%) had a history of cigarette smoking with an average year quit of 11.0 ± 9.2 years and 923 (54.3%) were actively smoking. For the 1699 baseline CTs, ILA were present in 41 (2.4%), indeterminate in 101 (5.9%) and not present in 1,557 (91.5%) (Table 1).

**Table 1:** Baseline characteristics

	(N = 1699)
Age	62.6 ± 6.24
Male sex	956 (56.3%)
Caucasian	1670 (98.3%)
BMI	29.2 ± 5.97
Actively smoking	923 (54.3%)
Pack years	48.5 ± 22.9
Years quit	11.1 ± 9.3
Years follow up	5.67 ± 1.59
Emphysema	982 (57.8%)
**Baseline lung-RADS®**	
0	1 (0.06%)
1	947 (55.7%)
2	560 (33.0%)
3	129 (7.6%)
4	62 (3.7%)
**Baseline qualitative ILA**	
No ILA	1557 (91.6%)
Indeterminate ILA	101 (5.9%)
ILA	41 (2.4%)

Interobserver agreement for presence of ILA with the indeterminate category included was moderate with κ = 0.42. With the indeterminate category combined with ILA yes category, interobserver agreement for presence of ILA increased (κ = 0.54).

### Pulmonary referral and ILD diagnosis

Of the 41 individuals with ILA on the baseline CT, 7 (17.1%) had seen a pulmonologist in the preceding 5 years (Table 2). 29 of the remaining 34 (85.3%), were referred to a pulmonologist after the baseline CT with a mean time to referral of 2.37 ± 2.87 years. For these 29 individuals, the primary reasons for pulmonary referral were nodules (12 patients), interstitial lung abnormalities (9 patients), COPD (3 patients), and other diagnoses (5 patients). ILD was diagnosed in 10 (24.4%) of the 41 patients with ILA on baseline CT with a mean time to diagnosis of 4.47 ± 2.72 years (Table 2).

**Table 2:** Pulmonary referral and ILD diagnosis

(N = 41)	N (%)	Years to referral or diagnosis
Followed by Pulmonary Pre-baseline CT scan	7 (17.1)	–
Referral to Pulmonary Only after baseline CT scan N = 34	29 (85.3)	2.37 ± 2.87
ILD diagnosis after baseline CT scan	10 (24.4)	4.47 ± 2.72

### Primary outcomes

#### All-cause mortality

There were 136 (8.0%) deaths during the study period (Table 3). 11 (26.8%) individuals with ILA, 13 (12.9%) with indeterminate ILA, and 112 (7.2%) individuals without ILA on baseline exams died during the study period. Causes of death are outlined in Additional file 2: Supplemental Table 2.

**Table 3:** Primary Outcomes by ILA Category

Primary outcomes	No ILA N = 1557 (91.6%)	Indeterminate ILA N = 101 (5.9%)	ILA N = 41 (2.4%)
Mortality	112 (7.2%)	13 (12.9%)	11 (26.8%)
Lung cancer	91 (5.8%)	8 (7.9%)	5 (12.2%)
Hospitalization	589 (37.8%)	44 (43.6%)	19 (46.3%)

On multivariable modeling (Table 4), the presence of ILA remained a significant predictor of death when adjusted for age, sex, BMI, pack years and active smoking, HR 3.87 (2.07, 7.21; p < 0.001). Due to the small number of ILA cases in our population, mortality was also analyzed with ILA and indeterminate groups combined and the presence of any ILA remained a significant predictor of death (Table 4).

**Table 4:** ILA Association with Mortality

	HR	p-value
No ILA	Reference group	–
Indeterminate ILA	1.84 (1.03, 3.27)	0.039
ILA	3.87 (2.07, 7.21)	< 0.001
Any ILA*	2.41 (1.57, 3.77)	< 0.001

*Multivariable analysis adjusted for age, sex, BMI, pack years, current smoker*

*Includes ILA + indeterminate ILA

**Fig. 2:**
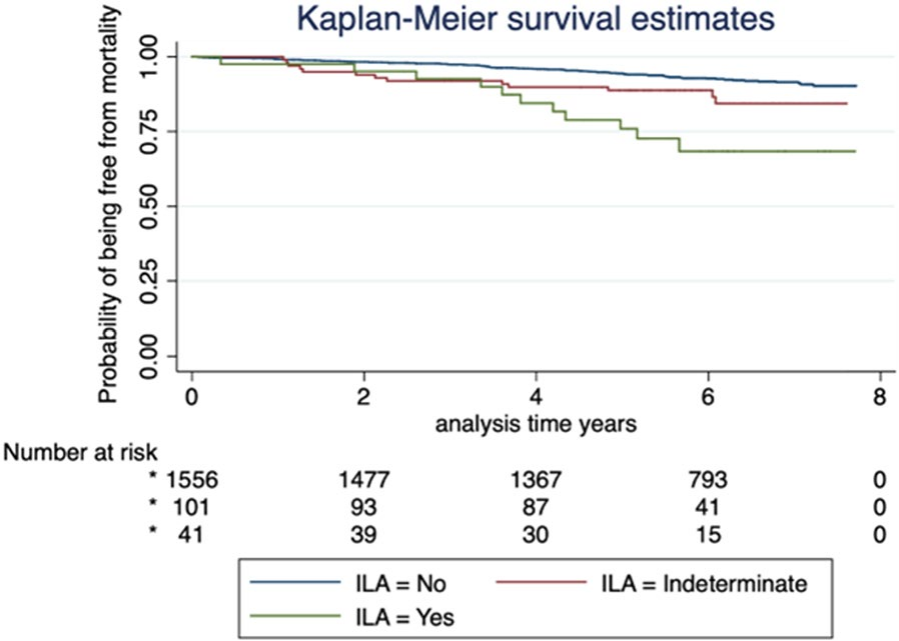
Kaplan–Meier Curve for Mortality and ILA

Figure 2 represents the Kaplan–Meier survival curve for the presence of ILA and mortality.

#### Hospital admissions

There were 1,574 all-cause hospital admissions in total during the study period; 652 (38.4%) were admitted to the hospital at least once (Table 3). Indeterminate ILA, ILA and any ILA were not significantly associated with all-cause hospital admission, HR 1.22 (0.90, 1.66; p = 0.198), HR 1.27 (0.80, 2.01; p = 0.304) and HR 1.24 (0.95, 1.61; p = 0.110), respectively, after adjusting for age, sex, BMI, pack years and active smoking.

#### Incidence of lung cancer

104 (6.12%) developed lung cancer during the study period (Table 3). The stage of lung cancer at diagnosis is described in Supplemental Table 3 (Additional file 2). Indeterminate ILA, ILA, and any ILA were not significantly associated with increased risk of lung cancer, HR 1.26 (0.61, 2.62;  = 0.527), HR 2.04 (0.82, 5.04; p = 0.124), and HR 1.48 (0.82, 2.66; p = 0.191), respectively, when adjusted for age, sex, BMI, pack years and active smoking.

#### Progression

There were 652 individuals (38.4% of total cohort) that had a T4 CT scan, of which 15 (2.3%) had baseline (T0) CT scans with ILA. From that group, 3 (20%) had resolution of ILA, 5 (33.3%) had stable findings, and 7 (46.7%) had progression on the T4 scan. Of the 3 individuals who had resolution of ILA, none died during the study period. In addition, of the 9 participants referred to pulmonary for ILA (described above), 4 had T4 scans available of which 3 (75%) had definite progression.

Of the 35 (5.4%) with indeterminate ILA at baseline, there were 7 (20.0%) that progressed to ILA, 15 (43.0%) remained indeterminate and 13 (37.1%) resolved. Of the remaining 602 (92.3%) with no ILA, there were 10 (1.7%) that progressed to ILA and 30 (5.0%) that progressed to indeterminate ILA (Additional file 1: Fig S1).

### Secondary outcomes

#### ILA phenotypes and UIP

The 41 participants with baseline ILA were scored for ground-glass opacities: 27 (65.5%); traction bronchiectasis: 18 (43.9%); reticular opacities: 41 (100%); honeycombing: 18 (43.9%); and cysts: 1 (2.4%). In addition, ~ 70% of individuals with ILA had emphysema on baseline CT (Additional file 2: Table S4). Both T0 and T4 scans were scored for UIP based on ATS criteria. Of the 41 (2.4%) baseline CTs and 29 (4.4%) longitudinal CTs with ILA, 4 (9.8%) and 6 (20.6%) were categorized as probable or typical UIP (Table 5).

**Table 5:** Usual interstitial pneumonia on baseline and longitudinal CT scans

	Baseline CT (n = 41) N (%)	T4 CT (n = 29) N (%)
Typical UIP	2 (4.9)	3 (10.3)
Probable UIP	2 (4.9)	3 (10.3)
Indeterminate UIP	23 (56.1)	18 (62.1)
Alternate Diagnosis	14 (34.2)	5 (17.2)

For 9 of these 10, the clinical reports documented the presence of fibrosis or interstitial lung disease, however none of the reports documented that a UIP pattern was present (Additional file 2: Table S5). Of the 4 patients with typical or probable UIP pattern on their baseline scan, 3 (75%) died, 2 (50%) developed lung cancer and 3 (75%) were admitted to the hospital during the study period. As of the date of censoring, none of the individuals had been initiated on anti-fibrotic therapy.

## Discussion

In this study, we found that interstitial lung abnormalities identified on CTLS exams were associated with increased mortality. Such association had been demonstrated in a number of other previous studies on ILA, and our study in the CTLS population is consistent with those previous reports. Despite finding a lower prevalence of ILA in the current study, the association of mortality (HR 3.87) was similar to that in the various cohorts investigated by Putman et al. [5] where the risk of mortality ranged from HR 1.3–2.7.

Our study demonstrated two additional key findings. First and most importantly, there remains a significant delay to diagnosis of ILD including progressive fibrotic ILD or IPF. Though most subjects with ILA in this cohort were referred to a pulmonologist after their baseline CT, the time to referral was on the order of years and the majority of patients were not referred for ILA but rather lung nodules. Similarly, in the quarter of individuals with ILA who were subsequently diagnosed with ILD during the study period, the mean time to diagnosis was nearly five years with earliest time to diagnosis of 1.14 years. When we specifically reviewed subjects categorized as typical or probable UIP by CT, 5 of the 10 patients did not receive a clinical diagnosis of ILD during the study period. Of the remaining 5, the mean time to diagnosis was 4.7 years. This long diagnostic delay could have been due to a number of factors including delay in referral, delay in recognizing ILA as ILD, and lack of symptoms at time of initial pulmonology evaluation. A recent study from Denmark evaluated reasons for diagnostic delay in IPF patients. They reported a median delay of 2.1 years and found that the largest contributors were delayed referral to a pulmonologist and subsequently to an ILD center [26]. Another study using the IPF-PRO registry demonstrated a much shorter time to diagnosis of a median 3.5 months from first imaging evidence of IPF but a median 13.6 months from symptom-onset [19]. The latter study highlights that the presence and availability of CT imaging may be the rate-limiting factor in IPF diagnosis.

Second, our study also demonstrated that almost half of individuals with ILA have progression on follow-up CT scans. Other studies also have shown significant rates of progression, varying from 20% in the NLST [27] to nearly 73% in the AGES-Reykjavik cohort [4]. This is consistent with the idea that many of the patients with ILA actually have ILD, and in some cases ILD that is early in the disease course. Further work can better define the impact of ILA progression and elucidate risk factors for progression such that those patients can be followed more closely or even initiated on antifibrotic therapy to prevent clinical worsening.

The findings of significant diagnostic delays and high incidence of progression highlight the need and opportunity to develop a system to identify individuals with ILD and bring them to specialist care earlier. CTLS programs may provide an opportunity to accomplish that goal as the CTLS population are at higher risk of ILD given their history of heavy smoking and serial CTs allow early identification of a progressive phenotype. Additionally, given the high prevalence of emphysema in this population, there may be a higher risk of combined pulmonary fibrosis and emphysema (CPFE) and the potential for worse outcomes [28].

This study has several limitations. Although it is a large cohort, it is a single-center study and our sample is not ethnically diverse, which reflects the patient population of our center. This lack of diversity may limit the generalizability of our study. Another limitation is that our analysis was limited to the in-network participants due to lack of follow-up data in the out-of-network cohort (~ 80% of participants) for some primary outcomes (hospitalizations, pulmonary referral) and covariates. Our hospitalization data was also limited to in-network hospitalizations, so hospitalizations outside our network were likely missed. This most likely resulted in an underestimation of the true hospitalization rates. Further, the percentage of participants with ILA in our study was small which may have limited our ability to determine some significant associations (e.g., risk of lung cancer and hospitalization). There may be several reasons for the lower prevalence of ILA in our study compared to other CT lung screening cohorts. In the study by Jin, et al., ILA prevalence was nearly 10% [27]. However, they included CTs with centrilobular nodularity and GGOs, which have since been eliminated from the ILA definition [3]. Brown, et al., also using the NLST cohort, found an ILA prevalence of 20% [7]. Their ILA criteria were less well-defined, including scarring, and thus may have included CTs that would be categorized as indeterminate or no ILA in our study based on Fleischner methodology [3]. In a recent study by Lee, et al., using criteria in line with Fleischner guidelines, the prevalence of ILA was 3% and of equivocal (or indeterminate) ILA was 4% [29]. These findings are similar to the prevalence in our study of 2.4% and 5.9%, respectively. The variable prevalence in ILA studies likely reflects the subjective nature of the 5% threshold for ILA and the patient population being studied (with older populations having been shown to have greater prevalence). In addition, our longitudinal analysis of progression was limited due to only about 1/3 of participants having T4 scans. Therefore, our results of progression incidence may not reflect the incidence in the entire cohort or in other similar populations. The number of T4 scans also limited our ability to analyze the data for risk factors of progression. Progression is an area of keen interest with both lung cancer screening and non-screening CTs providing an opportunity to identify patients with potential ILD before onset of symptoms (often late in the course). Future work is planned that will encompass a multi-center effort to better determine clinical impact and predictors of progression. Finally, the interobserver agreement determined by κ calculation was only moderate in our study. This may reflect the challenge of determining which patients met the 5% (of lung parenchymal involvement) threshold which in itself is arbitrary. Indeed, when eliminating the indeterminate category, which contains scans that may have had CT abnormalities but did not meet the 5% threshold, and including those patients in the ILA category, our interobserver agreement improved. There is limited data in the literature on interobserver agreement in visual analysis of ILA on CT scans, partly due to differing reading methodologies. Lee et al. reported a κ > 0.80 in their study of a general health screening cohort [29]. Jin et al. [27] reported a κ of 0.60 in their study of nearly 900 baseline NLST CTs for presence of ILA, a result which also demonstrates the need for more reproducible methodology. As discussed in the Fleischner Society ILA position paper [3], an important future direction in assessment of ILA is the development and validation of quantitative analysis [30–32]. Quantitative CT analysis using automated quantification of CT density may provide an avenue to achieve better reproducibility. In addition, the 5% threshold that defines ILA is arbitrary at this point. Other studies, including ours, have demonstrated that indeterminate scans, some of which are labeled such because they fall under the 5% threshold, are also associated with significant clinical outcomes such as mortality. This finding provides an opportunity to better define and validate the threshold that determines significant disease.

## Conclusions

This large CTLS cohort study demonstrated that ILA are significantly associated with mortality, confirming results from previous studies. We also observed a clinically significant time lag in the eventual diagnosis of ILD in this population, many of whom had progressive disease including IPF. Our data suggest CTLS programs may be an ideal setting to identify early disease and provide an opportunity to design clinical trials focused on early identification and treatment aimed to prevent disease progression.

## Supplementary Information

**Additional file 1: Figure S1.** Flowchart of study population and distribution of ILA progression on T4 scans.

**Additional file 2: Table S1.** Characteristics of in-network vs out-of-network participants. **Table S2.** Cause of death based on ILA categorization. **Table S3.** Lung cancer stage at diagnosis. **Table S4.** Presence and degree of emphysema in ILA patients. **Table S5.** Clinical CT reports of patients with definite or probable UIP.

## Data Availability

All data generated or analyzed during this study are included in the published article.

## Abbreviations

CTLS: CT lung screening
ILA: Interstitial lung abnormalities
ILD: Interstitial lung disease
IPF: Idiopathic pulmonary fibrosis
PFT: Pulmonary function test
UIP: Usual interstitial pneumonia
