# Supplementary material for: Interstitial lung abnormalities in a large clinical lung cancer screening cohort: association with mortality and ILD diagnosis
Source: Respir Res. 2023 Feb 14;24:49. doi: 10.1186/s12931-023-02359-9 (PMC9926562; doi:10.1186/s12931-023-02359-9)
Supplement: Supplementary file 1 — Additional file 1: Figure S1. Flowchart of study population and distribution of ILA progression on T4 scans. [file 12931_2023_2359_MOESM1_ESM.docx]

**Additional file 1**

**Figure S1. Flowchart of Study Population and Distribution of ILA Progression on T4 Scans**
