# Supplementary material for: Interstitial lung abnormalities in a large clinical lung cancer screening cohort: association with mortality and ILD diagnosis
Source: Respir Res. 2023 Feb 14;24:49. doi: 10.1186/s12931-023-02359-9 (PMC9926562; doi:10.1186/s12931-023-02359-9)
Supplement: Supplementary file 2 — Additional file 2: Table S1. Characteristics of in-network vs out-of-network participants. Table S2. Cause of death based on ILA categorization. Table S3. Lung cancer stage at diagnosis. Table S4. Presence and degree of emphysema in ILA patients. Table S5. Clinical CT reports of patients with definite or probable UIP. [file 12931_2023_2359_MOESM2_ESM.docx]

**Additional file 2**

**Table S1. Characteristics of In-Network vs Out-of-Network Participants**

|  | **In-Network**  **(N=1699)** | **Out-of- Network**  **(N=857)** |
| --- | --- | --- |
| Age | 62.6 ± 6.24 | 61.5± 5.87 |
| Male Sex | 956 (56.3%) | 450 (52.5%) |
| Caucasian | 1670 (98.3%) | 753 (87.9%) |
| BMI | 29.2 ± 5.97 | 28.3 ± 5.58 ***** |
| Actively Smoking | 923 (54.3%) | 359 (41.9%) |
| Pack Years | 48.5 ± 22.9 | 49.5 ± 21.2 |
| Years Quit | 11.1 ± 9.3 | 11.2 ± 8.2 |
| Years Follow Up | 5.67 ± 1.59 | 3.31 ± 2.45 ****** |
| Emphysema | 982 (57.8%) | 481 (56.1%) |
| Mortality | 136 (8.0%) | 34 (5.0%) ****** |
| Admission | 652 (38.4%) | 78 (11.5%) ****** |
| Cancer | 104 (6.1%) | 23 (3.4%) ****** |

***** 373 (43.5%) missing BMI data

** Based upon 679/857 out-of-network with follow-up data, compared to 1698/1699 in-network

**Table S2. Cause of Death Based on ILA Categorization**

| **ILA** | N=11 |
| --- | --- |
| Cancer | 2 (18.2%) * |
| Cardiac | 0 (0%) |
| Pulmonary | 4 (36.4%) |
|  | Respiratory Failure: 1 of 4 |
| Other | 5 (45.5%) |
| **Indeterminate ILA** | N=13 |
| Cancer | 5 (38.5%) * |
| Cardiac | 0 (0%) |
| Pulmonary | 1 (7.7%) |
|  | Respiratory Failure: 0 of 1 |
| Other | 7 (53.9%) |
| **No ILA** | N=112 |
| Cancer | 31 (27.7%) * |
| Cardiac | 17 (15.2%) |
| Pulmonary | 18 (16.1%) |
|  | Respiratory Failure: 7 of 18 |
| Other | 46 (41.1%) |

***** Of the total 38 (27.9%) cancer-related deaths, 21 (15.4% of all deaths) were directly attributed to lung cancer

**Table S3. Lung Cancer Stage at Diagnosis**

| **Cancer Histology** | **Limited/unknown** | **Stage 0-1** | **Stage 2** | **Stage 3** | **Stage 4** |
| --- | --- | --- | --- | --- | --- |
| Neuroendocrine -SCLC (n=10) | 7 (70%) | - | - | - | - |
| NSCLC (n=94) | 1 (1.1%) | 73 (77.7%) | 7 (7.4%) | 7 (7.4%) | 6 (6.4%) |

**Table S4. Presence and Degree of Emphysema in ILA Patients**

| **ILA** | **N=41** |
| --- | --- |
| No Emphysema | 9 (22.0%) |
| Mild Emphysema | 14 (34.1%) |
| Moderate Emphysema | 12 (29.3%) |
| Marked Emphysema | 3 (7.3%) |
| Not Scored | 3 (7.3%) |

**Table S5. Clinical CT Reports of Patients with Definite or Probable UIP**

| **Baseline CT** | **UIP category** | **Clinical CT scan report** |
| --- | --- | --- |
| 1 | DEFINITE | Progressed fibrotic interstitial lung disease question DIP |
| 2 | DEFINITE | Fibrotic interstitial lung disease with differential to include NSIP and DIP |
| 3 | PROBABLE | Fibrosis and nodularity |
| 4 | PROBABLE | Early fibrotic interstitial lung disease |
| **T4 CT** |  |  |
| 1 | DEFINITE | Fibrotic lung disease |
| 2 | DEFINITE | Infectious inflammatory process |
| 3 | DEFINITE | Mild fibrotic interstitial lung disease |
| 4 | PROBABLE | Fibrotic interstitial lung disease |
| 5 | PROBABLE | May reflect early Fibrotic interstitial lung disease |
| 6 | PROBABLE | Fibrotic interstitial lung disease |
